# Essential Role of the 14q32 Encoded miRNAs in Endocrine Tumors

**DOI:** 10.3390/genes12050698

**Published:** 2021-05-08

**Authors:** Lilla Krokker, Attila Patócs, Henriett Butz

**Affiliations:** 1 Department of Laboratory Medicine, Semmelweis University, H-1089 Budapest, Hungary; krkkr.lilla@gmail.com (L.K.); patocs.attila@med.semmelweis-univ.hu (A.P.); 2 Hereditary Cancers Research Group, Hungarian Academy of Sciences-Semmelweis University, H-1089 Budapest, Hungary; 3 Department of Molecular Genetics, National Institute of Oncology, H-1122 Budapest, Hungary

**Keywords:** miRNA, 14q32, miRNA cluster, DLK1-MEG3 locus, endocrine tumor, pituitary adenoma, adrenocortical cancer, neuroendocrine tumor, pheochromocytoma, thyroid cancer

## Abstract

Background: The 14q32 cluster is among the largest polycistronic miRNA clusters. miRNAs encoded here have been implicated in tumorigenesis of multiple organs including endocrine glands. Methods: Critical review of miRNA studies performed in endocrine tumors have been performed. The potential relevance of 14q32 miRNAs through investigating their targets, and integrating the knowledge provided by literature data and bioinformatics predictions have been indicated. Results: Pituitary adenoma, papillary thyroid cancer and a particular subset of pheochromocytoma and adrenocortical cancer are characterized by the downregulation of miRNAs encoded by the 14q32 cluster. Pancreas neuroendocrine tumors, most of the adrenocortical cancer and medullary thyroid cancer are particularly distinct, as 14q32 miRNAs were overexpressed. In pheochromocytoma and growth-hormone producing pituitary adenoma, however, both increased and decreased expression of 14q32 miRNAs cluster members were observed. In the background of this phenomenon methodological, technical and biological factors are hypothesized and discussed. The functions of 14q32 miRNAs were also revealed by bioinformatics and literature data mining. Conclusions: 14q32 miRNAs have a significant role in the tumorigenesis of endocrine organs. Regarding their stable expression in the circulation of healthy individuals, further investigation of 14q32 miRNAs could provide a potential for use as biomarkers (diagnostic or prognostic) in endocrine neoplasms.

## 1. Introduction

MicroRNAs (miRNAs) are single-stranded, small (~17–22 nucleotide long), protein non-coding RNA molecules that regulate gene expression post-transcriptionally by RNA interference. According to the canonical miRNA biogenesis, the mature miRNA is generated from a hairpin RNA precursor molecule produced by RNA polymerase II or III [1].

After biogenesis, the mature miRNA incorporates into a protein complex called miRISC (miRNA-induced silencing complex) [2]. In the miRISC complex miRNAs lead to translational repression, mRNA destabilization or mRNA cleavage through miRNA-mRNA interaction via base complementarity. MiRNAs target mRNAs mainly at 3′ untranslated regions but even the coding sequence or 5′UTR have been described to be miRNA target regions [2]. Recently, it has been discovered that in some particular cases miRNAs can even enhance gene expression [2].

Approximately 30–50% of all protein-coding genes are thought to be controlled by miRNAs [3]. As one miRNA targets several transcripts, and one mRNA is regulated by numerous miRNAs, the net physiological outcome is the result of a miRNA-target network. The role of miRNAs is primarily considered to set the gene expression to an optimal level as an adaptive process called “fine tuning” [4].

MiRNAs have been shown to be involved in the control of many physiological and pathophysiological processes, such as proliferation, differentiation, metabolism and apoptosis through the modulation of target gene expression. Altered miRNA expression has been identified in several endocrine diseases including neoplasms [5,6]. Depending on their target molecules, miRNAs are considered as oncomiRs or tumor suppressor miRNAs, and therefore they are often considered potentially useful biomarkers. MiRNAs are highly tissue-specific, and they may be unique identifiers of certain tumor types, even having different effects in different cell/tissue types.

The 14q32 miRNA cluster is among the largest polycistronic clusters comprising almost a hundred small non-coding RNAs, including a significant number of miRNAs [7]. MiRNAs located in this region cover over 5% of the known human miRNA genes [8,9].

**Figure 1:**
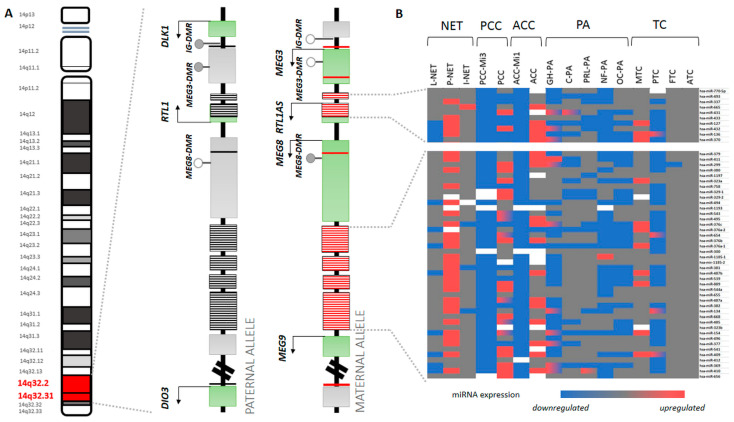
The imprinted 14q32 region. (**A**) On the paternal and maternal alleles middle: exons colored by grey indicate imprinted genes, exons colored by green indicate transcriptionally active state. DMR status is presented by grey (methylated) and white (unmethylated) circles. Black stripes represent imprinted miRNA genes, red stripes represent transcriptionally active miRNA genes. (**B**) On the heatmap (right): Representative expressional change of 14q32 miRNAs in endocrine tumors (downregulation and overexpression are presented compared to physiological, monoallelic expression. Heatmap was constructed using the data of 59 studies (see details in the methods section)). MiRNAs indicated on the heatmap are listed in Table 1 indicating the exact chromosomal localization. Colors indicate expression (blue color was used for low and *red* for high expression, grey represents monoallelic expression, *white*: no data available).

The 14q32 region is called the *DLK1-DIO3* domain or *DLK1-MEG3* cluster. Indeed, this cluster contains protein-coding (Delta-like 1 (*DLK1*), Deiodinase Iodothyronine Type III (DIO3) and Retrotransposon-like Gene 1 (*RTL1*)) and nonprotein-coding genes (ncRNAs, such as Maternally Expressed Gene 3 (*MEG3*), Maternally Expressed Gene 8 (*MEG8*) and *RTL1* antisense (*RTL1-AS*)), small nucleolar RNAs (snoRNAs) and miRNAs. This approximately 300 kilobase miRNA region can be divided into two parts: cluster A and cluster B. Cluster A includes *MEG3* and *RTL1* genes, while cluster B can be found 5′ from *MEG9* and *DIO3* genes (Figure 1A).

As miRNA expression can be influenced by several factors, Goossens and colleagues investigated the effect of the most common confounding factors: sex and age on the miRNA expression profile of this region [10]. The finding that 14q32 miRNA expression did not differ between men and women, and that no correlation with age was observed, highlighted the importance of this miRNA cluster in cell biology [10]. This was further supported by others who described that the 14q32 maternally imprinted locus was a major source of longitudinally stable circulating miRNAs as measured by small RNA sequencing of healthy individuals [11]. In addition, the serum level of 14q32 miRNAs was not significantly affected by common confounders such as age, body mass index (BMI) or time of centrifugation, nor alternative methods of expression data normalization [11].

14q32 miRNAs are frequently described deregulated in human diseases and cancers [12,13,14]. In line with the finding that miRNAs are highly tissue-specific [7,15], 14q32 miRNAs are considered both oncogenic and tumor suppressing depending on cell type [14,16].

The expression of 14q32 genes is regulated by genomic imprinting. The differential expression of alleles inherited from a mother or father at the genomic imprinted loci is realized by different methylation. The regulatory loci of the methylated nucleotides are called differentially methylated regions (DMRs). Differential methylation patterns at DMRs provide monoallelic expression from either maternal or paternal allele. Generally, imprinted genes have a key role in regulating growth and other physiological functions during embryonic development. Germline deletions and uniparental disomy of this locus in humans associate with developmental abnormalities and dysmorphism, suggesting that the 14q32 locus might have significant importance in development [17,18,19,20,21,22,23,24]. As several maternally imprinted genes limit growth during development, they usually possess a tumor-suppressor role in human cancer [25]. In line with this, the *DLK1-MEG3* cluster was frequently affected by allelic loss or epigenetic changes in various cancers [26,27,28,29]. In the 14q32 region, the paternally expressed protein coding genes are *DLK1*, *DIO3* and *RTL1* [30]. From the maternal allele *MEG3*, *MEG8* and *RTL1-AS* long noncoding RNAs are expressed [9,31]. This imprinted gene expression of this locus is under the control of three DMRs [20,32]: a DMR located 11 kb upstream of *MEG3* (also called intergenic differentially methylated region, IG DMR), a DMR 1.3 kb upstream of the *MEG3* transcription start site (*MEG3*-DMR) and a DMR found in the *DLK1* promoter (*DLK1-*DMR) [33] (Figure 1A).

The *MEG3*-IG DMR, which is methylated on the paternal allele and unmethylated on the maternal allele, functions as the primary imprinting control region (ICR) for the entire locus during development [34], whereas *MEG3*-DMR serves as the principal regulator in adult tissues [20]. This imprinted methylation pattern provides the reciprocal expression of *DLK1* and *MEG3*.

Accordingly, 14q32 miRNAs are also involved in the imprinting regulation [7,35] and altered 14q32 miRNA expression have been described in several diseases including malignancies [36,37,38,39,40,41]. Several studies have shown downregulation of miRNAs from the 14q32 region in different types of cancer, such as ovarian, breast, prostate, bladder, osteosarcoma, and gastrointestinal stromal, with significant correlations to poor prognosis and aggressiveness [42,43,44,45,46,47,48]. The tumor suppressor role has been recognized in several of the downregulated 14q32 miRNAs through targeting key oncogenes in glioblastoma, neuroblastoma, metastatic lung cancer, hepatic cancer and rhabdomyosarcoma [25,49,50,51]. In contrast, miRNAs from the 14q32 region may act as oncogenes as well [52,53,54], suggesting that these miRNAs may have different biological roles depending on the tissue of origin and genetic background.

14q32 miRNAs also influence prognosis of various cancers. Oshima et al. presented that the expression of 14q32 miRNAs was a favorable prognostic factor in patients with metastatic cancer [41]. Based on studies of Lussier et al. 2011 and 2012, the term oligomiR was introduced [55,56]. There were miRNAs differentially expressed between patients with limited numbers and slow progression of metastases (oligometastases) compared to patients with widely disseminated or rapidly progressive metastatic disease [55,56]. Interestingly, miRNAs encoded in the 14q32 were significantly enriched among oligomiRs [22]. Additionally, 14q32 miRNAs suppressed lung and liver metastases and correlated with improved clinical outcomes. In osteosarcoma 14q32 miRNAs also had prognostic significance, as an inverse correlation was described between aggressive tumor behavior (increased metastatic potential and accelerated time to death) and the residual expression of this miRNA locus [47].

Despite of the relevance of 14q32 miRNAs in other malignancies, regarding endocrine tumors, there has no comprehensive review published about the role of 14q32 miRNA cluster. In this work, the authors aimed to collect high-throughput miRNA studies performed in endocrine tumor samples, to extract the role and potential relevance of 14q32 miRNAs through investigating miRNA targets and to integrate the knowledge provided by literature data and bioinformatics predictions.

## 2. Materials and Methods

Literature mining was performed using Pubmed database using the following keywords: “14q32” or “miRNA” or “DLK1-MEG3” and combined with either of “endocrine tumors”, “neuroendocrine tumor”, “pituitary adenoma”, “adrenocortical tumor”, “pheochromocytoma” or “thyroid cancer”. Publication focused on 14q32 miRNAs regarding endocrine tumors, and high-throughput miRNA profiling studies of endocrine tumors were selected to construct an expression heatmap (Figure 1B, Table 1). Downregulated/overexpressed miRNAs were included when at least one study reported it significantly without conflict (conflict was considered when another study reported the opposite change). When conflicting information was observed between studies, gradient colour was used. Unfortunately, as in many studies, raw data were not available and only the significant lists were reported; only “downregulated” and “overexpressed” characteristics could be considered irrespectively of fold change. The heatmap itself was generated in Microsoft Excel (Microsoft Office Professional Plus 2013).

Also, miRbase MIMAT IDs of dominant mature 14q32 miRNAs were used for bioinformatics analysis (http://www.mirbase.org/, access date: 25 February 2021). Pathway analysis for 14q32 miRNA targets by gene set enrichment analysis for KEGG pathways was performed using DIANA TOOLS miRPath v.3 following target prediction by microT-CDS algorithm (http://snf-515788.vm.okeanos.grnet.gr/, access date: 25 February 2021). Gene ontology was assessed using miRDB Target Ontology Analysis module by selecting enrichment for Biological Process and Molecular Function (http://mirdb.org/ontology.html, access date: 25 February 2021).

## 3. miRNAs in Endocrine Tumors

### 3.1. Neuroendocrine Tumors (NET)

Neuroendocrine tumors (NETs) are a group of heterogeneous neoplasms arising from neuroendocrine cells throughout the body (most commonly from the gastrointestinal system or lungs). Although gastro-entero-pancreatic NETs (GEP-NETs) represent less than 1% of all digestive system cancers it consists 7–21% of all neuroendocrine neoplasms [113]. Lung NETs originate from pulmonary neuroendocrine cells accounting for approximately 25% of primary lung neoplasms and they are classified into typical carcinoids (TCs, well differentiated, low-grade), atypical carcinoids (ACs, well-differentiated, intermediate-grade), large cell neuroendocrine carcinomas (LCNECs, poorly differentiated, high-grade) and small cell lung cancer (SCLCs, poorly differentiated, high-grade) subtypes [114].

In GEP-NETs tissue, however, several miRNA studies have been published [115,116], scarce information was available regarding 14q32 miRNA profiles. In the study by Jiang et al., 29 overexpressed miRNAs derived from 14q32 were identified in insulinomas vs. normal islets, and several showed high abundance in insulinoma cells [64,117]. Unfortunately, this finding was not reported by others [65]. MiRNAs of this region were associated with prognosis, since miR-485–3p encoded at 14q32 region was significantly elevated in the metastatic tumors compared to the primary pancreatic NETs (pNET) (Table 2) [66]. Investigating small intestine (siNET) and colorectal NET, studies did not identify differentially expressed miRNAs encoded at 14q32 [118,119,120,121]. However, similar to pNET, miRNAs were linked to progression as 14q32 encoded miR-494 was significantly overexpressed in metastases compared to primary siNET [68]. Interestingly, by using miRNA expression profiling, Yoshimoto et al. described a similar pattern of miRNAs of carcinoids of the lung and gastrointestinal NETs, which was different from adenocarcinomas, small cell lung cancers and normal mucosal cells, suggesting a common origin of systemic carcinoids/NETs [57]. Also, miR-494 was downregulated in carcinoid vs. adenocarcinoma/normal tissue group [57]. Regarding lung NET types, Rapa et al. detected several 14q32 miRNAs (miR-409-3p, miR-409-5p, miR-376a, miR-376b, miR-381) upregulated in typical compared to atypical lung carcinoids [60]. Measured on 46 lung carcinoid tumors, a more extensive list of miRNAs expressed from 14q32 cluster detected as downregulated compared to their adjacent normal tissue pair samples by Deng et al. (miR-487b, miR-410, miR-369, miR-376a, miR-432, miR-409, miR-494, miR-136, miR-370, miR-127 and miR-154) [59].

Several studies evaluated the role of miRNAs as prognostic markers [116]. Among others, miR-409-3p, miR-409-5p, miR-431-5p, miR-411, miR-485 and miR-539 encoded at 14q32 were significantly downregulated in metastatic carcinoids compared to non-metastatic lung NETs, while miR-409-3p, miR-409-5p and miR-431-5p were found downregulated in cases with vascular invasion [60,116].

Also among 14q32 miRNAs, the expression of miR-127, miR-136, miR-154, miR-485, miR-770-5p showed negative correlation with tumor biology of lung NET, and miR-377* was identified, showing a significant impact on survival time [58].

A recent study reported that among the most abundant miRNAs in lung NET types, miR-127 encoded at 14q32 showed high expression in typical carcinoids tumors [61]. Besides, no 14q32 miRNA was identified as discriminatory miRNAs characteristic to typical carcinoid, atypical carcinoid, small cell lung cancer or large cell neuroendocrine carcinomas in the study of Wong et al. [61].

### 3.2. Pheochromocytoma-Paraganglioma (PPGL)

The rare pheochromocytomas (PCC) and paragangliomas (PGL) (together PPGL, incidence 1–8:1,000,000) arise from the same type of neural crest tissue of the sympathetic and parasympathetic paraganglia [122]. While tumors of the adrenal medulla are called PCCs, neoplasms developing from the head and neck, thoracic, abdominal or pelvic regions paraganglia are referred as PGLs. These tumors are usually benign and the 10-year overall survival is around ~96%, but 10% of PCC and even 40% of PGL occur as metastatic disease resulting in a 5-year survival below 50% [122]. Interestingly, PPGL has an extremely high rate of genetic susceptibility, when a germline mutation leads to autosomal dominant genetic syndromes (multiple endocrine neoplasia type 2A and 2B caused by *RET* mutations, von Hippel Lindau syndrome due to *VHL* mutations, neurofibromatosis type 1 with *NF1* mutations or hereditary PG syndrome caused by mutations of succinate dehydrogenase (SDH) genes, PPGL genes including *KIF1b*, *PHD2*, *TMEM127*, *MAX*, *FH*, *MDH2*, *GOT2* and *SLC25A11* [123]. Unfortunately, there are neither clear histopathological signs of malignant behavior or efficient therapy for malignant PPGL. Therefore, miRNAs have been good candidates being potential biomarkers.

Expectedly, miRNA profile in different genetic subtypes was also distinct and based on a high-throughput miRNA profiling [70,72,124] several 14q32 miRNAs were dysregulated in PPGL [71]. 14q32 encoded miR-493* was commonly downregulated in all molecular subtypes based on germline mutation [71]. The 14q32 miRNA profile (20 miRNAs) showed significant downregulation in *MAX*-related PPGLs and a subset of sporadic PC samples as well [71]. In *TMEM127*-related cases overexpression of eight 14q32 miRNAs were detected [71]. MiR-541 was found significantly overexpressed in *VHL*-related PCCs vs. sporadic counterparts, and these miRNAs had a lower level of expression in recurrent tumors compared to primary PCC [70]. As hypermethylation of *DLK-MEG3* locus was reported in approximately 10% of PCC samples [69], the pathogenic role of downregulated miRNAs located here was also proposed [125]. Indeed, in a comprehensive multi-omic approach, miRNA profiling by next generation sequencing (NGS) revealed 7 homogeneous subgroups of PCC. PCC samples of the Mi1,2 and Mi4-7 clusters exhibited higher 14q32 miRNA expression compared to Mi3 [69], while Mi3 subgroup was characterized by a strong silencing of the imprinted *DLK1-MEG3* cluster. In this study 15 of 17 tumors belonging to cluster Mi3 displayed loss-of-heterozygosity (LOH) at the 14q32 locus harboring *DLK1-MEG3*. The authors hypothesized that the loss of the maternal unmethylated allele might explain the repression of this imprinted miRNA cluster that was also supported by the methylation analysis of *MEG3* promoter [69]. Interestingly, PCC samples belonging to this Mi3 cluster were not associated with any germline mutation (they were all sporadic tumors) and they belonged to a distinct mRNA expression cluster (C2B) [69]. In line with these results, another large-scale study found the upregulation of miR-154, miR-337-3p in a subset of metastatic PCC compared to non-metastatic cases [73]. The downregulation of miR-409-3p, miR-369-3p was also identified in a different subset of metastatic tumors compared to benign ones [73]. In another study comparing benign and malignant cases, miR-431 was detected as upregulated in malignant tumors [72].

### 3.3. Adrenocortical Tumors

In aldosterone producing adenomas (APA), no differentially expressed miRNAs encoded at 14q32 were detected [126,127,128]. Interestingly, another study identified miR-410 and miR-433 as Wnt/β-catenin signaling regulatory miRNAs with significantly different expression between APA and peritumoral adrenal tissues using microarray [129]. A study investigating APA compared to non-APA adrenal tumors (adrenocortical adenoma (ACA), subclinical hypercortisolism (SH), non-functioning adrenal adenoma (NF)) identified miR-370 as overexpressed in aldosterone producing tumors. Also, similar to pheochromocytoma, in APA miRNA signature was also reflected in germline mutation carrier status [130], and miR-299 from locus 14q32 was found downregulated in *KCNJ5* mutant APA vs. non-*KCNJ5* mutant samples [130].

Regarding adrenocortical carcinoma (ACC), a combined genomic approach classified tumor samples into 3 clusters (Mi1-3) based on miRNA expression pattern [76]. ACC samples in cluster Mi1 showed the largest miRNA expression differences relative to normal adrenal samples. Samples in this Mi cluster were characterized by the downregulation of 38 miRNAs expressed from 14q32 locus and by the upregulation of miRNAs belonging to the Xq27.3 miRNA cluster [76]. By using SNP array and DNA methylation analysis, this study identified LOH of chromosome arm 14q in all Mi1 ACC tumors associated with *MEG3* promoter methylation. The authors suggested that the loss of the maternal unmethylated allele resulted in silencing of the 14q32 miRNA cluster in Mi1 ACC tumors, suggesting that this region had a key role in ACC pathogenesis [76]. Özata and his colleagues, however, found 5 miRNAs from 14q32 (miR-136, miR-127-3p, miR-487b, miR-376c and miR-432) overexpressed in ACC compared to normal adrenal cortex, but no 14q32 miRNAs were identified in association with survival [75]. Additionally, miR-376a and miR-376b overexpression were also described in ACC vs. ACA samples [54]. Interestingly, while miR-376a was detected as downregulated miRNA, miR-299-5p and miR-485-5p were found overexpressed in ACC vs. hormonally nonfunctioning adenoma, cortisol-producing adenoma and normal adrenal cortex [74].

Regarding ACC behavior miR-370, miR-376a, miR-376b, miR-376c, miR-377, miR-379, miR-382, miR-411, miR-487a, miR-494, and miR-495 encoded at 14q32 miRNA cluster were downregulated in non-aggressive ACC as compared to aggressive ones [54]. In another study, miRNA-665 was overexpressed in ACC as compared to benign adrenocortical tumors [131]. MiR-431 was also reported to be underexpressed in patients with ACC with progressive disease undergoing adjuvant therapy (mitotane, chemotherapy, and radiotherapy) compared to therapy responders [132]. Restoration of miR-431 increased cell responses to adjuvant therapy and led to cell cycle arrest at S phase. Authors demonstrated that Zinc Finger E-Box Binding Homeobox 1 (*ZEB1*), a target of miR-431, was implicated in reversal of the epithelial-mesenchymal transition (EMT), leading to increased cell responses to adjuvant therapies in ACC [132].

Interestingly, *DLK1* was found as a marker of adrenal gland tumor, which was in line with findings that 14q32 miRNAs (except for Mi1 subgroup) were upregulated in ACC suggesting a common transcriptional regulation of the entire locus in ACC (Figure 1) [133].

### 3.4. Pituitary Neuroendocrine Tumors (PitNET)

Pituitary adenomas are among the most frequent intracranial tumors with a high incidence rate of approximately 10–15% [134]. Although the great majority of them are benign, they represent significant morbidity by mass effect or by hormonal disturbance. Generally, pituitary adenomas are sporadic; only 5% of them occur as part of genetic syndromes such as MEN1, MEN4, Carney complex or McCune-Albright syndrome. Interestingly, miRNAs has been extensively investigated in pituitary tumors, including not only expressional reports, but functional studies [6]. As more than one hundred original publications have reported, findings have been extensively summarized by excellent reviews [6,135,136]. Here, authors aimed to only highlight the role of 14q32 miRNAs in pituitary adenomas using high-throughput studies comparing pituitary adenoma samples to normal pituitary tissues.

Prolactinomas. In a work using next generation sequencing, no 14q32 miRNAs were reported differentially expressed in prolactinomas [82]. However, with a targeted approach, Cheunsuchon et al. found 7 of 18 investigated 14q32 miRNAs in prolactin (PRL)-secreting tumors significantly down-regulated [81]. In line with these results, D’Angelo et al. detected downregulated miR-432 in PRL adenoma tissues and using functional in-vitro assays, high-mobility group AT-hook 2 mRNA (*HMGA2)* proved to be a miR-432 target [78]. On the contrary, Chen et al. detected miR-432 and miR-493 upregulation compared to normal anterior pituitary gland samples; moreover, they reported a significant positive correlation between the expression of the two miRNAs and the serum level of prolactin [87]. Additionally, of the 14q32 miRNA cluster, miR-410 was found to be upregulated in prolactinomas [86], as well as in 6 out of the 12 GH-secreting adenomas. This finding suggested that a reduced miR-410 expression seemed to be restricted to gonadotroph adenomas.

Growth hormone (GH) producing tumors. Numerous underexpressed miRNA located at 14q32 were identified using NGS and PCR array [80,82] (Figure 1). However, this was not entirely supported by other studies [77,78,81]. While some reported overexpression of miR-136 in GH-producing adenomas based on microarray profiling [77], a recent study using NGS and Bottoni et al. reported its downregulation compared to normal pituitary [79,82]. Nevertheless, miR-411-3p was overexpressed and miR-381 with miR-654-3p were downregulated from 14q32 locus [77,82]. MiR-370-3p was detected to be underexpressed in pituitary adenomas compared to normal pituitary and in non-functional pituitary adenomas (NFPA) compared to functional ones [137]. Furthermore, its level showed correlation with GH expression determined by immunohistochemistry [137]. Palumbo et al. identified 17 miRNAs to be differentially expressed in GH-producing pituitary tumors; however, none were encoded at 14q32 [138]. Pituitary tumor-transforming 1 (*PTTG1*) was identified as a target of miR-126 and miR-381 encoded at 14q32 cluster [139]. Also, Liang et al. demonstrated that overexpression of 4 14q32 miRNAs (miR-655, miR-300, miR-381 and miR-329) inhibited proliferation, migration and invasion, but induced apoptosis in GH3 and MMQ rat pituitary cells and regulated the *PTTG1* expression [90]. The authors suggested a negative feedback loop between *PTTG1* targeting miRNAs, *PTTG1* and p53 where p53 transcriptionally activated the expression of the four miRNAs, while *PTTG1* inhibited the transcriptional activity of p53 [90]. Among 14q32 miRNAs the downregulated miR-432 inhibited cell proliferation of GH3 cells and has a negative role on the growth regulation of pituitary adenoma by targeting *HMGA2* [78].

Non-functioning pituitary adenomas (NFPA). Several miRNAs mapped to 14q32 showed significant underexpression compared to normal pituitary in NFPA detected by different platforms [86,88,89]. Besides 32 downregulated miRNAs, *MEG3* and *DLK1* also showed underexpression in NFPA samples [89]. Indeed, another study investigating the silencing of the imprinted *DLK1-MEG3* locus in human NFPAs [81] also reported numerous 14q32 encoded miRNAs expression as lost or significantly diminished compared to normal pituitary. Furthermore, the authors identified these miRNA expression alterations together with increased methylation of *MEG3-*IG DMR [81,140,141]. This was in line with the finding that *MEG3* was not expressed in NFPAs; therefore the authors suggested that the silencing of the *DLK1-MEG3* locus played an important role in human NFPA pathogenesis [81,140,141]. Among 14q32 miRNAs miR-1185-1-3p was identified upregulated, while miR-493 downregulated [82]. Bottoni et al. found that miR-154, miR-127 and miR-134 were downregulated in NFPA and were predictive miRNAs for the histotype [79]. Others identified downregulation of miR-432 encoded at 14q32 in NFPA and gonadotroph adenomas [90]. The functional role of miR-432 was investigated in HP75 human pituitary adenoma cells, and miRNA transfection led to a significant reduction of cell number compared to controls [78]. Regarding gonadotroph adenomas reduced miR-410 expression seemed to be restricted to gonadotroph adenomas compared to other adenoma types [86]. Authors validated cyclin B1 (*CCNB1*) as target of miRNA-410 since its overexpression reduced *CCNB1* at protein and mRNA levels, decreasing cell proliferation.

Corticotroph adenomas. Cheunsuchon et al. investigated 18 members of the 14q32 miRNA cluster, among which several miRNAs identified as significantly downregulated (miR-127-3p, miR-136, miR-154, miR-299-5p, miR-329, miR-369-3p, miR-369-5p, miR-376c, miR-377 and miR-433) and only miR-431 was found overexpressed in tumors compared to normal tissues [81]. Although more than a few downregulated miRNAs were detected in adrenocorticotropin (ACTH)-secreting tumors, their expression levels were considered significantly higher compared to those found in NFPAs [81]. Accordingly, *DLK1* was found downregulated in corticotroph tumors [81]. While Stilling et al. detected 5 other miRNAs significantly downregulated located at 14q32 (miR-323-5p, miR-136*, miR-411*, miR-431*, miR-493) in corticotroph adenomas [85], others failed to detect any differentially expressed miRNAs from 14q32 region [84].

Pituitary carcinomas. In corticotroph carcinomas, miR-323-5p was downregulated in comparison to normal pituitary, and miR-493 was upregulated in carcinoma vs. adenoma [85]. It was suggested that miRNA-493 interacted with galectin-3 (*LGALS3,* lectin, galactoside-binding, soluble, 3) and Runt-related transcription factor 2 (*RUNX2*) genes, [142,143,144,145]. These data also showed that galectin-3 had a role in regulating cell proliferation and apoptosis of pituitary cells.

Pituitary oncocytoma. In pituitary oncocytoma, numerous underexpressed miRNAs (40% of all downregulated miRNAs) compared to normal control were mapped to 14q32 region [91].

### 3.5. Thyroid Carcinoma

Thyroid cancer is the most frequent malignant endocrine tumor. The majority of them (~95%) arise from follicular cells and classified as papillary (PTC, 75–80%), follicular (FTC, 10–15%) or anaplastic thyroid cancer (ATC, 0.2–2%) [146]. Tumors developing from calcitonin secreting parafollicular C cells are a distinct entity, and called medullary thyroid cancer (MTC) representing ~5–10% of all thyroid cancers [146]. This subtype commonly occur sporadically; however, approximately 10–25% of them are hereditary and appear as part of MEN2 syndrome, caused by germline mutations of the *RET* proto-oncogene [146]. Most of the well differentiated thyroid cancer (including PTC, FTC) has excellent prognosis; however, patients with ATC have 6–12 months median survival [147].

Nikiforova et al. detected markedly different profiles of miRNA expression between MTC and all other thyroid tumors that derives from follicular cells, reflecting tissue-specific characteristics of miRNAs [94]. Among these, several 14q32 miRNAs were overexpressed in MTC compared to normal and other thyroid cancer types [94]. Expectedly, Lassalle et al. detected numerous miRNAs differentially expressed between sporadic and hereditary MTC cases including miR-136, miR-487b, miR-376a,c, and miR-127 located at 14q32 miRNA cluster [92]. Interestingly, the highly expressed miR-375 was revealed as a novel circulating prognostic marker for MTC patients as well, as MTC patients had significantly higher miR-375 plasma levels than healthy controls and subjects in remission [148]. Additionally, high circulating miR-375 level was associated with significantly reduced overall survival and was a strong prognostic factor of poor prognosis [148].

Numerous miRNAs were described in non-medullary thyroid cancer types, however, with controversial results. Therefore, a comprehensive re-analysis integrating 21 thyroid cancer miRNA studies by Saiselet et al. determined the commonly reported differentially expressed miRNAs in non-medullary thyroid carcinomas compared to normal tissues [149]. Of the investigated studies, in FTC and ATC, no differentially expressed miRNA encoded at 14q32 miRNAs occurred except the downregulated miR-299 in FTC [149,150]. However, 4 overexpressed (miR-134, miR-136, miR-409, miR-654) and several underexpressed (miR-124, miR-134, miR-300, miR-379, miR-382 and miR-494-3p, miR-494-5p and miR-495) 14q32 miRNA were identified in PTC samples. In a more recent study, the global downregulation of miRNAs from the 14q32 region in human PTC was also confirmed [95]. The decreased miR-654-3p levels with long-term PTC progression in Tg-Braf mice was also observed and the level of miR-654-3p inversely correlated with epithelial-mesenchymal transition (EMT) [95]. The in-vitro restoration of miR-654-3p inhibited cell proliferation and migration and induced reprogramming of metastasis-related genes, supporting the tumor suppressor role for this miRNA [95]. Interestingly, in another study analyzing miRNA expression profiles in classical-type PTC, follicular-variant PTC, and tall-cell variant, no 14q32 miRNA was detected compared normal adjacent thyroid tissues [96].

From a clinical point of view miRNAs are suggested as potential biomarkers, as cytology following fine-needle aspiration biopsy (FNAB) are interpreted as indeterminate without definitive diagnosis regarding thyroid tumors in 3–6% to 10–25% [147]. However, miRNAs located at 14q32 did not help in discriminating benign vs. malignant thyroid lesions from FNAB samples [6].

## 4. Different Expression of 14q32 miRNA Cluster Members

14q32 locus contains more than forty miRNAs, and previously it had been thought that they were generated from one polycistronic transcript containing the whole miRNA cluster under a coordinated regulation with the *MEG3* non-coding RNA located upstream [7,12,151]. Also, hyper-methylation of the 14q32 DMRs was described to associate with decreased 14q32 miRNA expression and vice versa, suggesting that the entire imprinted cluster is regulated jointly [37,44,45,152].

However, in several endocrine tumors, the pattern of 14q32 miRNAs were not so homogenous. Indeed, in other tissues and tumor types, similar findings were described [10,153,154]. Also, in non-tumorous cells not all of the members of 14q32 miRNA cluster were expressed in all tissues and 14q32 miRNAs demonstrated varying level of expression, suggesting other possible regulating mechanisms [155]. Indeed, the expression of protein-coding and non-coding genes encoded at the 14q32 locus was regulated by epigenetic changes, but the exact mechanism behind controlling this process is not entirely known [21,36,46,68,156,157].

However, several mechanisms have been identified in the context of this variable expression of the cluster members, among which *methylation* was the most obvious. Genomic imprinting imbalance could result in the differential modulation of paternally and maternally expressed genes from the 14q32 region that might serve as an explanation, at least in part, for the increased levels of *DIO3* observed in some papillary thyroid cancer samples [95,153,158]. Various DNA methylation patterns of the 14q32 locus were observed in different blood vessel types, which were not associated with miRNA expression [10]. Direct correlation was not possible to be proven between 14q32 estimated methylation fraction of multiple cytosines followed by guanine residues (CpG) in the 3 DMRs located along 14q32 and 14q32 miRNA expression [10]. Moreover, neither DNMT gene expression or DNA methylation did not correlate with primary or mature miRNA expression [10]. In urothelial carcinoma, distinctive epigenetic alterations were again observed at the three regions controlling *DLK1* and *MEG3* expression [154]. The authors suggested that altered nucleosomal positioning could account for the irregular patterning of DNA methylation; namely, that one specific CpG site became significantly hypomethylated in cancer cells, while methylation of flanking sites rather increased [154].

Recent studies have shown that *chromatin remodeling by lncRNA-mediated mechanisms*, may also participate in regulating the expression of the 14q32-encoded miRNAs [46,68].

Additionally, Greife and colleagues demonstrated the loss of active and gain of repressive histone modifications at all regulatory sequences using chromatin immunoprecipitation [154].

Differences in miRNA *splicing, primary transcript processing or pre-miRNA cleavage and maturation* were also reported related to 14q32 miRNAs [159]. Some suggested that the expression of miRNA clustered on 14q32 might be particularly sensitive to changes in the miRNA biogenesis pathway [159,160,161]. A large proportion of 14q32 encoded miRNAs contained structural features associated with Dicer-independent processing [162], therefore Ago2-dependent pre-miRNA processing [162,163] was particularly important for the biogenesis of miRNA in this cluster. Goossens et al. reinforced that miRNA-specific expression fingerprints implied individual regulation of 14q32 miRNA expression [10].

*RNA Binding Proteins* (RBPs) were other post-transcriptional regulators of miRNA expression. RBPs bound precursor miRNAs and promoted or inhibited their maturation. For instance, Myocyte Enhancer Factor 2A (*MEF2A*) was such an RBP regulating miR-329 and miR-494 encoded at 14q32 chromosomal region [164]. Cold-inducible RNA-binding protein (*CIRBP*) and hydroxyacyl-CoA dehydrogenase trifunctional multienzyme complex subunit β (*HADHB*) were also RNA binding proteins that regulated 14q32 miRNA expression [165].

The different expressional pattern regarding this miRNA cluster was also observed by Manodoro et al., who attributed it to the presence of the binding sites of *CCCTC-binding factor (CTCF)* which was implicated in transcriptional activation/repression and imprinting [32,157,166]. CTCF exerted its regulatory function by binding to unmethylated DNA in an allele-specific manner [167,168]. Interestingly, it was found that different CTCF binding sites display a different influence on 14q32 miRNA expression depending on the position [157].

Altogether, these data suggest that multiple mechanisms other than genetic mutations or chromosomal loss might be involved in the regulation of 14q32-encoded miRNAs.

## 5. Functional Impact of 14q32 miRNAs

By analyzing function of individual 14q32 miRNAs, besides several molecular functions and biological processes, TGFβ and Wnt signaling were also identified, which are frequently involved in tumor development (Supplementary Table S1).

However, as 14q32 miRNAs more or less function in cooperation, we performed target prediction and gene set enrichment analysis to investigate the net effect of their co-expressional pattern. Several cancer-related pathways (including TGF-β signaling, Ras signaling, ErbB signaling), pathways involved in invasiveness and metastasis development (e.g., proteoglycans in cancer, adherens junction) or influencing pluripotency and stemness were identified as a potentially functional role (Table 3).

Literature/experimental data also suggested the regulation of axon guidance, actin cytoskeleton, focal adhesion, mammalian target of rapamycin, calcium, mitogen-activated protein kinase and ErbB signaling pathways by 14q32 miRNAs [159]. Liu et al. found that these miRNAs were highly associated with cellular pluripotency [35]. Interestingly, the transcription factor, *MEF2A* regulated the expression of 14q32 miRNAs being a direct target of miR-329 [169]. Uppal and colleagues, using mRNA profiling and bioinformatics, demonstrated that 14q32 miRNAs target genes in PI3K/AKT/mTOR and TGF-β pathways were involved in focal adhesion, cell–extracellular matrix interactions, gap junctions and actin cytoskeleton, resulting in impaired adhesion, invasion and migration, processes that were essential for the development of metastases [22,170,171]. The regulation of PI3K/AKT/mTOR and TGF-β pathways by 14q32 miRNAs was also strengthened by Qian and colleagues in hemopoietic stem cells as well [172].

In osteosarcoma, the decrease of 14q32 miRNA levels stabilized c-MYC protooncogene expression and consequently increased the level of oncogenic miR-17-92 miRNA cluster [173]. Cell cycle and epithelial-mesenchymal transition (EMT) were also proved to be influenced by 14q32 miRNAs [38,47,95,174]. Genes involved in metastasis development were also enriched among 14q32 miRNA targets [47]. Cyclin dependent kinase 5 (*CDK5*) and Twist Family BHLH Transcription Factor 1 (*TWIST1*) have been reported to increase metastasis through regulating cell cycle and EMT and they were found to be upregulated in osteosarcoma tumors with low levels of 14q32 miRNAs [47,174,175]. Furthermore, thymidine kinase 1 (*TK1*), that expressed at high levels in proliferating cells and appeared to correlate with high risk in multiple cancer types, was negatively correlated with 14q32 miRNA expression [47,176,177,178].

In thyroid cancer, the role of 14q32 miRNAs was particularly investigated. Geraldo et al. showed that 14q32 miRNAs contributed to tumor progression and metastasis by targeting key regulators of cell adhesion, migration, proliferation, hypoxic response and wound healing [95]. The reintroduction of miR-654-3p reversed EMT by targeting, hence increasing the expression of cadherin 1 (*CDH1*) and catenin α 1 (*CTNNA1*), and decreasing the expression of Snail Family Transcriptional Repressor 2 (*SNAI2*) [95]. Also, it was demonstrated that genes involved in tumor progression (ECM-remodeling and metastasis) were restored after transfection of a miR-654-3p mimetic [95].

Angiogenesis and neovascularization were also significantly regulated by 14q32 miRNAs. This was confirmed by identifying vascular endothelial growth factor A (*VEGFA*) as a target of miR-494 [169], as well as of miR-127 [179]. Furthermore, miR-495 were demonstrated to target C-C motif chemokine ligand 2 (*CCL2*), through which it affects proliferation and apoptosis of human umbilical vein endothelial cells (HUVECs) [180]. Forkhead box O1 (*FOXO1*) influencing endothelial growth and proliferation [181], wound closure and vascular density was also identified as a target molecule of miR-544 in colorectal cancer [181,182,183]. The role of 4 14q32 miRNAs was additionally proved in in vivo experiments, as the inhibition of miR-329, miR-494, miR-487b and miR-495 in mice stimulated neovascularization after hind limb ischemia [10,169].

## 6. Summary and Discussion

Altogether, 14q32 miRNAs have an important role in development and tumorigenesis. The importance of this miRNA cluster regulatory function is represented by the finding that their expression is stable in healthy cells, and even in cell-free serum samples. Their expression is also independent of the most common confounding factors, such as age, sex or BMI. Several studies have shown downregulation of miRNAs from the 14q32 region in different types of cancer; however, 14q32 miRNAs are overexpressed in some cancer types reflecting the tissue-specificity of miRNA function.

Among different endocrine tumors in pituitary adenoma and oncocytoma, papillary thyroid cancer and a particular subset of pheochromocytoma and adrenocortical cancer are characterized by the downregulation of almost all miRNAs encoded by the 14q32 cluster. In the subgroups of ACC and PCC, the silencing of the imprinted 14q32 cluster due to LOH of chromosome arm 14q or 14q32 locus. In other tumor types including NFPA and/or gonadotroph pituitary adenomas increased methylation of the region DMRs could explain the orchestrated downregulation of the coding and non-coding gene expression of the entire region.

Interestingly, pancreas NET, most of the adrenocortical cancer cases and medullary thyroid cancer are particularly distinct, as 14q32 miRNAs are overexpressed in these tumors. The role of these overexpressed miRNAs should be further investigated in relation to tumorigenesis.

In the third group of endocrine tumor types such as pheochromocytoma and growth-hormone producing tumors, and based on the expression pattern of 14q32 miRNAs, however, both increased and decreased expression of 14q32 miRNAs cluster members were observed. In the background of this phenomenon, methodological, technical and biological factors can be hypothesized as well. Different researcher groups applied different study design, different sample numbers, RNA extraction and miRNA quantification methods which all could lead to controversial results. Also, as detailed above, several biological explanations have been revealed in the context of the various expressional pattern of the different members of 14q32 miRNA clusters; e.g., different methylation pattern, chromatin remodeling, histone modifications, alteration of miRNA biogenesis, the effects of RNA binding proteins and transcription factors. These factors await being further investigated in pheochromocytoma and growth-hormone producing pituitary adenomas.

Unfortunately, the function of 14q32 in endocrine tumors is not so broadly investigated compared to other tumor types. However, in different types of pituitary adenoma cell lines, 14q32 miRNAs proved their tumor suppressor role by inducing cell cycle arrest and cell growth inhibition by targeting *PTTG1* and *HMGA2*. In PTC, the significance of another 14q32 miRNA, miR-654-3p, was demonstrated in in vivo experiments as it influenced proliferation, migration, metastasis-related gene expression and EMT.

14q32 miRNAs are also associated with disease prognosis. In endocrine tumors several 14q32 miRNAs were identified as prognostic markers in pancreatic, small intestinal and lung NET. Some of these miRNAs are also linked to patient survival in lung NET. In PCC 14q32 miRNAs indicated metastatic cases compared to non-metastatic cases, assisting the discrimination of benign and malignant tumors. Also, the higher expression of miRNAs encoded at 14q32 were associated with aggressive ACC cases. Finally, while in MTC, 14q32 miRNA miR-375 has not only been reported as a strong prognostic factor of poor prognosis, but its higher level was associated with reduced overall survival, while on the contrary, the decreased 14q32 miRNA in PTC was associated with long-term progression.

Based on data presented in Figure 1, we found some miRNAs unique among endocrine tumor types (miR-337 and miR-758 overexpression in pNET, miR-329 and miR-541 overexpression in PCC/PGL and miR-376c overexpression in MTC). However, there is no full consensus among miRNA profiling studies; i.e., not all studies identified the same miRNAs differentially expressed, and therefore these findings should be further validated. The discrepancy can be due to different study design, difference in sample number (statistical power) and also due to technical factors (e.g., different platforms for high-throughput profiling). Analyzing expressional pattern of 14q32 miRNA cluster instead of individual miRNAs, characteristic/unique expression profile was described in MTC compared to other thyroid carcinoma type [94] or in different types of pituitary adenoma [79,81]. Furthermore, even the same type of endocrine tumor, pheochromocytoma can be grouped by different miRNA pattern according to germline mutational background [71]. As 14q32 miRNAs were found to be dysregulated in several cancer types, globally, they cannot be considered as unique tissue biomarkers. Another level of use can be their application in liquid biopsy samples e.g., in circulation. This is supported by the finding that 14q32 miRNAs were stable in serum and their level was not significantly affected by common confounder factors [11]. Already, the potential use of circulating miRNAs has been suggested in endocrine tumors. In ACC a unique, tissue specific miRNA, miR-483-5p has been suggested as a potential candidate as predictive marker for recurrence [54], and miR-146a-5p and miR-221-3p as serum biomarkers for post-treatment monitoring of PTC patients [96]. Unfortunately, to our best knowledge, no 14q32 mapped miRNA in circulation has been investigated in endocrine tumors. However, in non-endocrine tumor types their expression indicate prognosis and survival [155]. Accordingly, this miRNA cluster was proved to influence EMT process and metastasis development [95,132], hence, they may be used as prognostic biomarker in endocrine tumors as well.

The evolutional role and constraint can be considered another point of view of imprinted miRNAs [184,185,186]. It was suggested that imprinted noncoding RNAs was under distinctive selective forces when regulating transcripts of the allele inherited from the other parent [184,185,186,187]. Accordingly, when an mRNA had sequence complementary to an imprinted miRNA, the complementary miRNA-mRNA sequences pair originated from different alleles. This can be considered a communication between the maternal and paternal alleles, hence the two alleles coordinate their activities [186]. The kinship theory considers genomic imprinting as a mechanism to change gene dosage, because it has a differential effect on the fitness of matrilineal and patrilineal relatives [184,185,186,187]. Additionally, Haig and Mainieri suggested that when an imprinted miRNA targets an unimprinted mRNA, their interaction may have different fitness consequences for the loci encoding the miRNA and mRNA [184]. In a recent study, *HMGA2* was proposed as an attractive candidate to be one of the original targets because its 3′ regulatory region contained multiple predicted target sites for 14q32 miRNAs, with some of these target sites evolutionarily older than the 14q32 miRNA cluster [184]. *HMGA2* have special role in this context as it has reported to be overexpressed in several cancer type including endocrine tumors and often regulated by miRNAs [188,189], which also highlights the role of 14q32 miRNAs in tumorigenesis.

## 7. Conclusions

Similar to other cancer types, 14q32 miRNAs have a significant role in the tumorigenesis of endocrine glands. In different endocrine tumor types this miRNA cluster reflects the general tissue specificity of miRNAs regarding expression pattern, tumor suppressor or oncogene function, and they have a significant impact on prognosis as well. Regarding the stable expression of 14q32 miRNAs in healthy individuals in circulation, the further investigation of this miRNA cluster could provide an option to use them as diagnostic or prognostic biomarkers in endocrine neoplasms.

## Figures and Tables

**Table 1:** MiRNA studies used for heatmap generation.

Tumor Type		Study	miRNA Profiling Platform
NET	Lung	Yoshimoto et al., 2018 [57]	microarray
		Mairinger et al., 2014 [58]	TaqMan array
		Deng et al., 2014 [59]	microarray
		Rapa et al., 2015 [60]	PCR array
		Wong et al., 2020 [61]	NGS
	Pancreas	Zimmermann et al., 2018 [62]	TaqMan array
		Roldo et al., 2006 [63]	microarray
		Jiang et al., 2015 [64]	PCR array
		Zhou et al., 2016 [65]	microarray (GSE43796) reanalysis
		Lee et al., 2015 [66]	Nanostring nCounter
	small intestinal	Yoshimoto et al., 2018 [57]	microarray
		Arvidsson et al., 2018 [67]	microarray
		Li et al., 2013 [68]	microarray
		Miller et al., 2016	Nanostring nCounter
PPGL		Castro-Vega et al., 2015 [69]	NGS
		Tömböl et al., 2010 [70]	TaqMan array
		de Cubas et al., 2013 [71]	microarray
		Meyer-Rochow et al., 2010 [72]	microarray
		Calsina et al., 2019 [73]	individual qPCR
ACC		Tömböl et al., 2009 [74]	TaqMan array
		Chabre et al., 2012 [54]	microarray
		Özata et al., 2011 [75]	microarray
		Assié et al., 2014 [76]	NGS
Pituitary	GH	Mao et al., 2010 [77]	microarray
		D’Angelo et al., 2012 [78]	microarray
		Bottoni et al., 2007 [79]	microarray
		Butz et al., 2009 [80]	TaqMan array
		Cheunsuchon et al., 2011 [81]	individual qPCR
		He et al., 2019 [82]	NGS
	ACTH	Gentilin et al., 2013 [83]	individual TaqMan assay
		Amaral et al., 2009 [84]	individual TaqMan assay
		Stilling et al., 2010 [85]	microarray
		Cheunsuchon et al., 2011 [81]	individual qPCR
	PRL	He et al., 2019 [82]	NGS
		Müssnich et al., 2015 [86]	microarray
		Chen et al., 2012 [87]	NGS
		Cheunsuchon et al., 2011 [81]	individual qPCR
	NFPA	He et al., 2019 [82]	NGS
		Darvasi et al., 2019 [88]	NGS, TaqMan array and microarray
		Butz et al., 2011 [89]	TaqMan array
		Liang et al., 2015 [90]	individual qPCR
		Cheunsuchon et al., 2011 [81]	individual qPCR
		Müssnich et al., 2015 [86]	microarray
		Bottoni et al., 2007 [79]	microarray
	OC	Krokker et al., 2019 [91]	NGS
Thyroid	MTC	Lassalle et al., 2016 [92]	microarray
		Hudson et al., 2013 [93]	Taqman array
		Nikiforova et al., 2008 [94]	Taqman array
	PTC	Geraldo et al., 2017 [95]	NGS (obtained from The Cancer Genome Atlas dataset)
		Rosignolo et al., 2017 [96]	Taqman array
		Tetzlaff et al., 2007 [97]	microarray
		Linwah et al., 2011 [98]	microarray
		Jacques et al., 2013 [99]	microarray
		Lassalle et al., 2011 [100]	microarray
		Mancikova et al., 2015 [101]	NGS
		Peng et al., 2014 [102]	microarray
		Riesco-Eizaguirre et al., 2015 [103]	NGS
		Saiselet et al., 2015 [104]	NGS
		Swierniak et al., 2013 [105]	NGS
	FTC	Nikiforova et al., 2008 [94]	TaqMan array
		Rossing et al., 2012 [106]	microarray
		Dettmer et al., 2013 [107]	Taqman array
		Jacques et al., 2013 [99]	microarray
		Lassalle et al., 2011 [100]	microarray
		Mancikova et al., 2015 [101]	NGS
		Wojtas et al., 2014 [108]	microarray
	ATC	Hébrant et al., 2014 [109]	microarray
		Visone et al., 2007 [110]	microarray
		Boufraqech et al., 2015 [111]	microarray
		Braun et al., 2010 [112]	microarray

ACC: adrenocortical carcinoma; ACTH: corticotroph adenoma; ATC: anaplastic thyroid cancer; FTC: follicular thyroid carcinoma; GH: growth hormone; MTC: medullary thyroid carcinoma; NET: neuroendocrine tumor; NFPA: nonfunctional pituitary adenoma; OC: oncocytoma; PPGL: pheochromocytoma-paraganglioma; PRL: prolactin; PTC: papillary thyroid carcinoma.

**Table 2:** Summary of the most important dysregulated, 14q32 encoded miRNAs in different endocrine neoplasms.

**NET**	
miR-485-3p	increased in the metastatic tumors compared to the primary pNET
miR-494	overexpressed in metastases compared to primary siNET
	downregulated in carcinoid vs. adenocarcinoma/normal lung tissue
miR-376a, miR-376b, miR-381, miR-409-3p, miR-409-5p,	upregulated in typical compared to atypical lung carcinoids
miR-127, miR-136, miR-154, miR-369, miR-370, miR-376a, miR-410, miR-432, miR-409, miR-487b, miR-494	downregulated in lung carcinoid compared to adjacent normal tissue
miR-409-3p, miR-409-5p, miR-411, miR-431-5p, miR-485 and miR-539	downregulated in metastatic carcinoids compared to non-metastatic lung NET
miR-127, miR-136, miR-154, miR-485, miR-770-5p	negative correlation with tumor biology of lung NET
**PPGL**	
miR-493-5p	commonly downregulated in all PCC molecular subtypes (based on germline mutation)
miR-127-3p, miR-136, miR-154-3p/5p, miR-323a-3p, miR-337-5p/-3p, miR-369-5p, miR-370, miR-376a-5p, miR-376c, miR-377, miR-382, miR-409-5p, miR-410, miR-485-3p és 5p, miR-487a, miR-495, miR-539, miR-543, miR-758, miR-889	downregulation in MAX-related PPGLs and a subset of sporadic PCC
miR-154-3p, hsa-miR-369-5p, hsa-miR-485-5p, hsa-miR-487a, hsa-miR-495, hsa-miR-543, hsa-miR-656, hsa-miR-889	overexpression in TMEM127-related PPGL cases
miR-541	overexpressed in VHL-related PCC vs. sporadic PCC, decreased expression in recurrent tumors compared to primary tumors
miR-154, miR-337-3p	upregulated in a subset of metastatic PCC compared to non-metastatic cases
miR-409-3p, miR-369-3p	downregulation in a subset of metastatic PCC compared to benign PCC
miR-431	upregulated in malignant tumors compared to benign
**Adrenocortical Tumors**	
miR-370	overexpressed in APA compared to non-APA adrenal tumors
miR-299	downregulated in KCNJ5 mutant APA vs. non-KCNJ5 mutant samples
14q32 miRNA cluster	whole miRNA cluster downregulation in Mi1 subset of ACC
miR-136, miR-127-3p, miR-487b, miR-376c and miR-432	overexpressed in ACC compared to normal adrenal cortex
miR-376a, miR-376b	overexpression in ACC vs. ACA
miR-376a	downregulated in ACC vs. NF adenoma, CPA and normal adrenal cortex
miR-299-5p, miR-485-5p	overexpressed in ACC vs. NF adenoma, CPA and normal adrenal cortex
miR-370, miR-376a, miR-376b, miR-376c, miR-377, miR-379, miR-382, miR-411, miR-487a, miR-494, miR-495	downregulated in non-aggressive ACC as compared to aggressive ones
miRNA-665	overexpressed in ACC as compared to benign adrenocortical tumors
miR-431	implicated in adjuvant therapy response in ACC
**PitNET**	
miR-127-3p, miR-154, miR-329, miR-337, miR-369-5p, miR-376c, miR-432, miR-433	downregulated in PRL adenoma vs. normal
miR-410	overexpressed in PRL adenoma vs. normal
miR-411-3p	overexpressed in GH adenoma vs. normal
miR-381, miR-654-3p	downregulated in GH adenoma vs. normal
miR-127, miR-134, miR-136, miR-154, miR-323a, miR-337, miR-369, miR-370, miR-376a-1, miR-376a-2, miR-376b, miR-376c, miR-379, miR-380, miR-381, miR-382, miR-409, miR-410, miR-411, miR-431, miR-432, miR-433, miR-487b, miR-493, miR-494, miR-495, miR-539, miR-543, miR-544a, miR-654, miR-656, miR-770-5p, miR-889	downregulated in NF adenoma vs. normal
miR-1185-1-3p	upregulated in NF adenoma vs. normal
miR-127-3p, miR-136, miR-154, miR-299-5p, miR-323-5p, miR-329, miR-369-3p, miR-369-5p, miR-376c, miR-377, miR-411-3p, miR-431-3p, miR-433, miR-493	downregulated in corticitroph adenoma vs. normal
miR-431, miR-493	overexpressed in corticotroph carcinoma vs. adenoma
miR-127, miR-136, miR-154, miR-299, miR-323a, miR-323b, miR-329-1, miR-329-2, miR-369, miR-370, miR-376a-1, miR-376a-2, miR-376b, miR-376c, miR-379, miR-381, miR-382, miR-409, miR-411, miR-431, miR-485, miR-487b, miR-494, miR-539, miR-654, miR-889	downregulated in oncocytoma vs. normal
**Thyroid Carcinoma**	
miR-9, miR-127, miR-136, miR-154, miR-323, miR-376a,c, miR-370, miR-487b	upregulated in MTC vs. normal
miR-299	downregulated in FTC
miR-134, miR-136, miR-409, miR-654	overexpressed in PTC
miR-134, miR-300, miR-379, miR-382, miR-494-3p, miR-494-5p, miR-495	downregulated in PTC
miR-654-3p	inverse correlation with PTC progression

ACA: adrenocortical adenoma; ACC: adrenocortical carcinoma; APA: aldosterone producing adenoma; CPA: cortisol producing adenoma; FTC: follicular thyroid carcinoma; GH: growth hormone; KCNJ5: potassium inwardly rectifying channel subfamily J member 5; MAX: MYC associated factor X; MTC: medullary thyroid carcinoma; NET: neuroendocrine tumor; NF: nonfunctional; PCC: pheochromocytoma; pNET: pancreatic neuroendocrine tumor; PPGL: pheochromocytoma-paraganglioma; PRL: prolactin; PTC: papillary thyroid carcinoma; siNET: small intestinal neuroendocrine tumor; TMEM127: transmembrane protein 127; VHL: von Hippel-Lindau tumor suppressor.

**Table 3:** Top 20 significant signaling pathway regulated by 14q32 miRNAs.

KEGG Pathway	*p*-Value	# Genes	# miRNAs
Hippo signaling pathway (hsa04390)	2.635 × 10^−7^	103	47
Proteoglycans in cancer (hsa05205)	2.507 × 10^−6^	132	48
Pathways in cancer (hsa05200)	3.424 × 10^−6^	255	48
Adherens junction (hsa04520)	1.345 × 10^−5^	57	41
TGF-β signaling pathway (hsa04350)	1.582 × 10^−5^	58	45
Axon guidance (hsa04360)	2.465 × 10^−5^	88	45
Rap1 signaling pathway (hsa04015)	3.946 × 10^−5^	141	48
Glioma (hsa05214)	4.825 × 10^−5^	47	43
Ras signaling pathway (hsa04014)	4.825 × 10^−5^	146	49
Circadian rhythm (hsa04710)	6.429 × 10^−5^	27	37
Lysine degradation (hsa00310)	9.643 × 10^−5^	33	43
Signaling pathways regulating pluripotency of stem cells (hsa04550)	0.0001	96	50
FoxO signaling pathway (hsa04068)	0.0001	92	46
Thyroid hormone signaling pathway (hsa04919)	0.0001	79	46
Ubiquitin mediated proteolysis (hsa04120)	0.0004	93	44
Dorso-ventral axis formation (hsa04320)	0.0006	24	36
Prion diseases (hsa05020)	0.0009	17	26
ErbB signaling pathway (hsa04012)	0.0011	63	45
Renal cell carcinoma (hsa05211)	0.0015	48	41
Pancreatic cancer (hsa05212)	0.0023	48	43

## Data Availability

All relevant data are included in the manuscript.

## Supplementary Materials

The following are available online at https://www.mdpi.com/article/10.3390/genes12050698/s1, Table S1: 14q32 miRNAs and their function.
