# Supplementary material for: Essential Role of the 14q32 Encoded miRNAs in Endocrine Tumors
Source: Genes (Basel). 2021 May 8;12(5):698. doi: 10.3390/genes12050698 (PMC8151414; doi:10.3390/genes12050698)
Supplement: Supplementary file 1 [file genes-12-00698-s001.zip › genes-1170076-supplementary.pdf]

Supplementary Table 1. 14q32 miRNAs and their function. Biological Process and Molecular Function were obtained from miRDB database Gene Ontology Module (http://mirdb.org/mirdb/ontology.html)

miRNA gene name	miRNA gene miRBase ID	Chromosomal band	Chromosomal location	miRNA cluster	mature miRNA name	mature miRNA miRBase ID	GO Biological Process	GO Molecular Function
hsa-miR-770	MI0005118	14q32.2	14:100852409-100852431	A	hsa-miR-770-5p	MIMAT0003948	chondroitin sulfate proteoglycan biosynthetic process, positive regulation of transcription by RNA polymerase II	DNA-binding transcription activator activity, RNA polymerase II-specific, RNA polymerase II cis-regulatory region sequence-specific DNA binding
hsa-miR-493	MI0003132	14q32.2	14:100869060-100869148	A	hsa-miR-493-5p	MIMAT0002813	inhibitory synapse assembly, synaptic transmission, GABAergic, endoplasmic reticulum tubular network organization, cell differentiation in hindbrain, presynapse assembly, forebrain neuron development, cerebellar cortex formation, regulation of ryanodine-sensitive calcium-release channel activity, Wnt signaling pathway, calcium modulating pathway, microtubule polymerization, adherens junction organization, homophilic cell adhesion via plasma membrane adhesion molecules,	LBD domain binding, benzodiazepine receptor activity, GABA-gated chloride ion channel activity, inhibitory extracellular ligand-gated ion channel activity, GABA-A receptor activity, beta-catenin binding, SMAD binding, PDZ domain binding, guanyl-nucleotide exchange factor activity, RNA polymerase II-specific DNA-binding transcription factor binding, transcription coactivator activity
					hsa-miR-493-3p	MIMAT0003161	nervous system development, cell differentiation, cell death in response to oxidative stress,	
hsa-miR-337	MI0000806	14q32.2	14:100874493-100874585	A	hsa-miR-337-5p	MIMAT0004695	histone mRNA catabolic process, pathway-restricted SMAD protein phosphorylation, positive regulation of nitric-oxide synthase activity, cellular response to nerve growth factor stimulus, positive regulation of receptor-mediated endocytosis, peptidyl-threonine phosphorylation, protein acylation, positive regulation of protein kinase B signaling, positive regulation of peptidyl-tyrosine phosphorylation,	activin-activated receptor activity, SMAD binding, protein homodimerization activity, RNA polymerase II cis-regulatory region sequence-specific DNA binding, DNA-binding transcription factor activity, RNA polymerase II-specific, metal ion binding,
hsa-miR-665	MI0005563	14q32.2	14:100875033-100875104	A	hsa-miR-665	MIMAT0004952	positive regulation of catabolic process, regulation of plasma membrane bounded cell projection organization, cell morphogenesis involved in differentiation, animal organ morphogenesis, nervous system development, regulation of developmental process, regulation of localization, regulation of signaling, regulation of cell communication, negative regulation of cellular process, regulation of biological quality, localization, positive regulation of cellular process	protein binding
hsa-miR-431	MI0001721	14q32.2	14:100881007-100881120	A	hsa-miR-431-5p	MIMAT0001625	-	protein binding
hsa-miR-433	MI0001723	14q32.2	14:100881886-100881978	A	hsa-miR-433-5p	MIMAT0026554	-	-
					hsa-miR-433-3p	MIMAT0001627	sphingolipid biosynthetic process, regulation of organelle organization, regulation of transcription by RNA polymerase II, regulation of localization, positive regulation of nitrogen compound metabolic process, positive regulation of cellular process,	transcription coregulator binding, RNA polymerase II cis-regulatory region sequence-specific DNA binding, DNA-binding transcription factor activity, RNA polymerase II-specific
hsa-miR-127	MI0000472	14q32.2	14:100882979-100883075	A	hsa-miR-127-5p	MIMAT0004604	homophilic cell adhesion via plasma membrane adhesion molecules, nervous system development	-
					hsa-miR-127-3p	MIMAT0000446	-	ion transmembrane transporter activity, phosphorylative mechanism
hsa-miR-432	MI0003133	14q32.2	14:100884483-100884576	A	hsa-miR-432-5p	MIMAT0002814	negative regulation of signal transduction, positive regulation of transcription by RNA polymerase II, regulation of cellular protein metabolic process, regulation of biological quality, system development	protein binding
					hsa-miR-432-3p	MIMAT0002815	positive regulation of cell projection organization, regulation of dendrite development, neuron projection morphogenesis, cell junction organization, inorganic cation transmembrane transport, cell migration, negative regulation of signaling, negative regulation of cell communication, negative regulation of response to stimulus, regulation of biological quality	nitric-oxide synthase regulator activity, metal ion transmembrane transporter activity
hsa-miR-136	MI0000475	14q32.2	14:100884702-100884783	A	hsa-miR-136-5p	MIMAT0000448	positive regulation of macromolecule biosynthetic process, regulation of transcription by RNA polymerase II, negative regulation of macromolecule metabolic process, negative regulation of cellular metabolic process, positive regulation of nitrogen compound metabolic process, regulation of signal transduction, cellular macromolecule metabolic process	nucleic acid binding
hsa-miR-370	MI0000778	14q32.31	14:100911139-100911213	A	hsa-miR-370-5p	MIMAT0026483	positive regulation of high voltage-gated calcium channel activity, animal organ morphogenesis, nervous system development	protein binding
					hsa-miR-370-3p	MIMAT0000722	protein phosphorylation, brain development, cell development, anatomical structure morphogenesis, negative regulation of cellular process, detection of chemical stimulus involved in sensory perception of smell	protein tyrosine kinase activity, protein binding
hsa-miR-379	MI0000787	14q32.31	14:101022066-101022132	B	hsa-miR-379-5p	MIMAT0000733	-	-
					hsa-miR-379-3p	MIMAT0004690	Wnt signaling pathway, calcium modulating pathway, synapse organization, regulation of synapse organization, modulation of chemical synaptic transmission, positive regulation of cellular component biogenesishead development, regulation of organelle organization, positive regulation of cellular component organization, regulation of multicellular organismal development, positive regulation of RNA metabolic process	protein binding
hsa-miR-411	MI0003675	14q32.31	14:101023325-101023420	B	hsa-miR-411-5p	MIMAT0003329	-	-

hsa-miR-299	MI0000744	14q32.31	14:101023794-101023856	B	hsa-miR-299-5p	MIMAT0002890	cardiac atrium development, ventricular septum development, aorta development, positive regulation of neuron apoptotic process, artery morphogenesis, cell morphogenesis involved in neuron differentiation, axon development, neuron projection morphogenesis	protein binding
					hsa-miR-299-3p	MIMAT0000687	-	protein binding
hsa-miR-380	MI0000788	14q32.31	14:101025017-101025077	B	hsa-miR-380-5p	MIMAT0000734	-	-
					hsa-miR-380-3p	MIMAT0000735	regulation of developmental process, detection of chemical stimulus involved in sensory perception of smell	protein binding
hsa-miR-1197	MI0006656	14q32.31	14:101025564-101025651	B	hsa-miR-1197	MIMAT0005955	Wnt signaling pathway, calcium modulating pathway, glycosaminoglycan biosynthetic process, intracellular receptor signaling pathway, negative regulation of protein phosphorylation, synaptic signaling, negative regulation of cell differentiation, neuron development	enzyme binding, ion binding
hsa-miR-323a	MI0000807	14q32.31	14:101025732-101025817	B	hsa-miR-323a-5p	MIMAT0004696	positive regulation of peptidyl-serine phosphorylation of STAT protein, natural killer cell activation involved in immune response, animal organ development, regulation of transcription, DNA-templated	-
					hsa-miR-323a-3p	MIMAT0000755	neuron projection extension, peptidyl-threonine phosphorylation,negative regulation of epithelial cell proliferation,cytokinesis, Golgi organization, stress-activated protein kinase signaling cascade, peptidyl-serine phosphorylation, locomotory behavior, response to transforming growth factor beta, regulation of mRNA metabolic process, negative regulation of intracellular signal transduction, response to peptide hormone, mRNA processing, regulation of neuron projection development, posttranscriptional regulation of gene expression, positive regulation of protein kinase activity, cell morphogenesis involved in differentiation, negative regulation of cell cycle	protein threonine kinase activity, protein serine kinase activity, magnesium ion binding, phosphatidylinositol binding, mRNA binding, ATP binding, protein binding
hsa-miR-758	MI0003757	14q32.31	14:101026020-101026107	B	hsa-miR-758-5p	MIMAT0022929	-	-
					hsa-miR-758-3p	MIMAT0003879	gonadal mesoderm development, circulatory system development, regulation of transcription by RNA polymerase II, positive regulation of cellular metabolic process, positive regulation of nitrogen compound metabolic process,	sodium ion transmembrane transporter activity, transcription regulator activity, metal ion binding
hsa-miR-329-1	MI0001725	14q32.31	14:101026785-101026864	B	hsa-miR-329-5p	MIMAT0026555	negative regulation of cellular process, innate immune response, detection of chemical stimulus involved in sensory perception of smell	metal ion binding, G protein-coupled receptor activity, olfactory receptor activity
hsa-miR-329-2	MI0001726	14q32.31	14:101027100-101027183	B	hsa-miR-329-3p	MIMAT0001629	dendrite morphogenesis, neuron projection extension, negative regulation of BMP signaling pathway, homophilic cell adhesion via plasma membrane adhesion molecules, peptidyl-serine phosphorylation, regulation of protein stability, regulation of cellular protein catabolic process, histone modification, axon development, positive regulation of cellular catabolic process, regulation of cell migration, regulation of growth, positive regulation of transcription by RNA polymerase II, central nervous system development, regulation of cell differentiation, positive regulation of cellular protein metabolic process	poly-purine tract binding, calcium ion binding, catalytic activity, acting on a protein, protein binding
hsa-miR-494	MI0003134	14q32.31	14:101029634-101029714	B	hsa-miR-494-5p	MIMAT0026607	peptidyl-amino acid modification	-
					hsa-miR-494-3p	MIMAT0002816	central nervous system neuron axonogenesis, circadian regulation of gene expression, regulation of dendritic spine development, negative regulation of cellular response to growth factor stimulus, limb development, histone modification, chromatin remodeling, developmental growth, regulation of neuron projection development, regulation of vesicle-mediated transport, positive regulation of organelle organization, brain development, positive regulation of transcription by RNA polymerase II, protein modification by small protein conjugation or removal, regulation of protein localization, negative regulation of transcription, DNA-templated, embryo development, positive regulation of cell differentiation, cell-cell signaling	enzyme binding, catalytic activity, acting on a protein, DNA binding, metal ion binding
hsa-miR-1193	MI0014205	14q32.31	14:101030052-101030129	B	hsa-miR-1193	MIMAT0015049	-	-
hsa-miR-543	MI0005565	14q32.31	14:101031987-101032064	B	hsa-miR-543	MIMAT0004954	cellular response to leukemia inhibitory factor, regulation of mRNA splicing, via spliceosome, homophilic cell adhesion via plasma membrane adhesion molecules, protein dephosphorylation, rhythmic process, proteasome-mediated ubiquitin-dependent protein catabolic process, blood vessel morphogenesis, cell morphogenesis involved in differentiation, regulation of neuron projection development, positive regulation of transcription by RNA polymerase II, negative regulation of transcription by RNA polymerase II, protein modification by small protein conjugation, positive regulation of cellular component organization, regulation of cellular catabolic process	ubiquitin conjugating enzyme binding, ubiquitin-like protein transferase activity, protein domain specific binding, metal ion binding, organic cyclic compound binding, heterocyclic compound binding
hsa-miR-495	MI0003135	14q32.31	14:101033755-101033836	B	hsa-miR-495-5p	MIMAT0022924	positive regulation of dendrite extension, cellular response to alcohol, negative regulation of gene expression, negative regulation of cellular macromolecule biosynthetic process, negative regulation of RNA metabolic process, positive regulation of RNA metabolic process, positive regulation of protein metabolic process, positive regulation of cellular biosynthetic process, positive regulation of macromolecule biosynthetic process, regulation of transcription by RNA polymerase II	protein binding
					hsa-miR-495-3p	MIMAT0002817	prostate gland growth, interleukin-6-mediated signaling pathway, lung epithelial cell differentiation, hippo signaling, myotube cell development, axis elongation, endocardial cushion development, regulation of dendritic spine development, regulation of filopodium assembly, protein monoubiquitination, regulation of dendritic spine morphogenesis, regulation of heart growth, regulation of dendrite developmentregulation of cell-matrix adhesion, regulation of circadian rhythm, Notch signaling pathway	transcription coactivator binding, ubiquitin conjugating enzyme binding, nuclear receptor activity, histone acetyltransferase activity, beta-catenin binding, histone deacetylase binding, SMAD binding, phosphoprotein phosphatase activity, histone binding, mRNA binding

hsa-miR-376c	MI0000776	14q32.31	14:101039690-101039755	B	hsa-miR-376c-5p	MIMAT0022861	-	lipid binding, protein binding
					hsa-miR-376c-3p	MIMAT0000720	synapse organization, axon guidance	-
hsa-miR-376a-2	MI0003529	14q32.31	14:101040069-101040148	B	hsa-miR-376a-2-5p	MIMAT0022928	-	-
hsa-miR-654	MI0003676	14q32.31	14:101040219-101040299	B	hsa-miR-654-5p	MIMAT0003330	SMAD protein signal transduction, cognition, skeletal system development, regulation of secretion by cell, regulation of protein localization, positive regulation of transcription by RNA polymerase IInervous system development, regulation of multicellular organismal process, regulation of signal transduction, detection of chemical stimulus involved in sensory perception of smell,	growth hormone receptor binding, enzyme binding
					hsa-miR-654-3p	MIMAT0004814	positive regulation of transcription by RNA polymerase II, cell development, sensory perception of chemical stimulus	DNA-binding transcription activator activity, RNA polymerase II-specific, RNA polymerase II cis-regulatory region sequence-specific DNA binding, metal ion binding, protein binding
hsa-miR-376b	MI0002466	14q32.31	14:101040436-101040535	B	hsa-miR-376b-5p	MIMAT0022923	-	protein binding
					hsa-miR-376b-3p	MIMAT0002172	nervous system development, regulation of primary metabolic process	ion binding, protein binding
hsa-miR-376a-1	MI0000784	14q32.31	14:101040782-101040849	B	hsa-miR-376a-5p	MIMAT0003386	-	protein binding
					hsa-miR-376a-3p	MIMAT0000729	nervous system development, regulation of primary metabolic process	ion binding, protein binding
hsa-miR-300	MI0005525	14q32.31	14:101041363-101041445	B	hsa-miR-300	MIMAT0004903	monoubiquitinated protein deubiquitination, suckling behavior, branch elongation of an epithelium, epithelial cell fate commitment, neuroblast proliferation, cell surface receptor signaling pathway involved in heart development, central nervous system projection neuron axonogenesis, peptidyl-lysine trimethylation, mRNA destabilization, positive regulation of histone methylation, blood vessel remodeling, microtubule polymerization, circadian regulation of gene expression, canonical Wnt signaling pathway	E-box binding, beta-catenin binding, mRNA binding, histone binding, RNA polymerase II-specific DNA-binding transcription factor binding, DNA-binding transcription activator activity, RNA polymerase II-specific, transcription coregulator activity, protein kinase binding
hsa-miR-1185-1	MI0003844	14q32.31	14:101042977-101043062	B	hsa-miR-1185-1-5p	MIMAT0005798	cGMP catabolic process, negative regulation of cell proliferation involved in kidney development, trans-synaptic signaling by endocannabinoid, cell-cell adhesion mediated by integrin, endothelial cell fate commitment, trans-synaptic signaling, modulating synaptic transmission, trachea morphogenesis, negative regulation of cAMP-mediated signaling, ganglion development, mammary gland epithelial cell differentiation, pharyngeal system development, beta-catenin-TCF complex assembly, salivary gland morphogenesis, positive regulation of cardiac muscle cell proliferation, epithelial tube branching involved in lung morphogenesis, cardiac epithelial to mesenchymal transition, positive regulation of mesenchymal cell proliferation	mRNA 3'-UTR binding, transmembrane receptor protein tyrosine kinase activitySMAD binding, beta-catenin binding, activating transcription factor binding, transcription corepressor activity, histone deacetylase binding, protein phosphatase binding, GTPase regulator activity, chromatin binding, protein serine/threonine kinase activity, RNA polymerase II cis-regulatory region sequence-specific DNA binding, identical protein binding, metal ion binding, G protein-coupled receptor activity
hsa-mir-1185-2	MI0003821	14q32.31	14:101044198-101044283	B	hsa-miR-1185-5p	MIMAT0005798	cGMP catabolic process, negative regulation of cell proliferation involved in kidney development, trans-synaptic signaling by endocannabinoid, cell-cell adhesion mediated by integrin, endothelial cell fate commitment, trans-synaptic signaling, modulating synaptic transmission, trachea morphogenesis, negative regulation of cAMP-mediated signaling, ganglion development, mammary gland epithelial cell differentiation, pharyngeal system development, beta-catenin-TCF complex assembly, salivary gland morphogenesis, positive regulation of cardiac muscle cell proliferation, epithelial tube branching involved in lung morphogenesis, cardiac epithelial to mesenchymal transition, positive regulation of mesenchymal cell proliferation, glutamate secretion, face morphogenesis, Golgi to plasma membrane protein transport, cAMP-mediated signaling, cellular response to amyloid-beta, mRNA stabilization	mRNA 3'-UTR binding, transmembrane receptor protein tyrosine kinase activitySMAD binding, beta-catenin binding, activating transcription factor binding, transcription corepressor activity, histone deacetylase binding, protein phosphatase binding, GTPase regulator activity, chromatin binding, protein serine/threonine kinase activity, RNA polymerase II cis-regulatory region sequence-specific DNA binding, identical protein binding, metal ion binding, G protein-coupled receptor activity
					hsa-miR-1185-3p	MIMAT0022713	cGMP catabolic process, negative regulation of cell proliferation involved in kidney development, trans-synaptic signaling by endocannabinoid, cell-cell adhesion mediated by integrin, endothelial cell fate commitment, trans-synaptic signaling, modulating	mRNA 3'-UTR binding, transmembrane receptor protein tyrosine kinase activitySMAD binding, beta-catenin binding, activating transcription factor binding, transcription corepressor activity, histone deacetylase binding, protein phosphatase binding, GTPase regulator activity, chromatin binding, protein serine/threonine kinase activity, RNA polymerase II cis-regulatory region sequence-specific DNA binding, identical protein binding, metal ion binding, G protein-coupled receptor activity
hsa-miR-381	MI0000789	14q32.31	14:101045920-101045994	B	hsa-miR-381-5p	MIMAT0022862	-	-
					hsa-miR-381-3p	MIMAT0000736	regulation of lymphoid progenitor cell differentiation, monoubiquitinated protein deubiquitination, pericardium morphogenesis, suckling behavior, branch elongation of an epithelium, epithelial cell fate commitment, neuroblast proliferation, cell surface receptor signaling pathway involved in heart development, regulation of RIG-I signaling pathway, central nervous system projection neuron axonogenesis, peptidyl-lysine trimethylation, mRNA destabilization, positive regulation of histone methylation, blood vessel remodeling, circadian regulation of gene expression, negative regulation of epithelial cell differentiation, cardiac ventricle morphogenesis, canonical Wnt signaling pathway	E-box binding, mRNA binding, histone bindingDNA-binding transcription activator activity, RNA polymerase II-specific, protein domain specific binding, protein-containing complex binding, catalytic activity, acting on a protein, metal ion binding
hsa-miR-487b	MI0003530	14q32.31	14:101046455-101046538	B	hsa-miR-487b-5p	MIMAT0026614	neuromuscular junction development, hormone-mediated signaling pathway, cellular response to steroid hormone stimulus, actin cytoskeleton organization, negative regulation of developmental process, regulation of cell differentiation, anatomical structure morphogenesis	RNA polymerase II transcription coactivator binding, nuclear receptor activity, enzyme binding
					hsa-miR-487b-3p	MIMAT0003180	-	-
hsa-miR-539	MI0003514	14q32.31	14:101047321-101047398	B	hsa-miR-539-5p	MIMAT0003163	dendrite morphogenesis, neural retina development, hippocampus development, peptidyl-serine phosphorylation, Wnt signaling pathway, vesicle organization, regulation of ion transport, regulation of ion transport	histone deacetylase binding, protein kinase binding, cytoskeletal protein binding, RNA polymerase II cis-regulatory region sequence-specific DNA binding, transcription regulator activity,metal ion binding

					hsa-miR-539-3p	MIMAT0022705	homophilic cell adhesion via plasma membrane adhesion molecules, nucleocytoplasmic transport, cellular protein localization, nervous system development, biological regulation	ion binding, protein binding
hsa-miR-889	MI0005540	14q32.31	14:101047901-101047979	B	hsa-miR-889-5p	MIMAT0026719	gonadal mesoderm development, regulation of neurotransmitter secretion, homophilic cell adhesion via plasma membrane adhesion molecules, hormone-mediated signaling pathway, regulation of ion transport, nervous system development, adaptive immune response, detection of chemical stimulus involved in sensory perception of smell	voltage-gated potassium channel activity, phosphatidylinositol bisphosphate binding, histone binding, calcium ion binding, DNA-binding transcription factor activity, RNA polymerase II-specific, sequence-specific double-stranded DNA binding
					hsa-miR-889-3p	MIMAT0004921	negative regulation of cytoplasmic translation, positive regulation of transcription from RNA polymerase II promoter involved in cellular response to chemical stimulus, negative regulation of mRNA catabolic process, regulation of sodium ion transport, epithelial cell proliferation, hormone-mediated signaling pathway, hormone-mediated signaling pathway, regulation of cation channel activity, myeloid cell differentiation, modulation of chemical synaptic transmission, protein phosphopantetheinylation, axon development,	SMAD binding, mRNA 3'-UTR binding, transferase activity
hsa-miR-544a	MI0003515	14q32.31	14:101048712-101048733	B	hsa-miR-544a	MIMAT0003164	positive regulation of cell-substrate junction organization, regulation of dendritic spine development, regulation of insulin receptor signaling pathway, homophilic cell adhesion via plasma membrane adhesion molecules, myelination, negative regulation of translation, positive regulation of supramolecular fiber organization, protein phosphorylation, positive regulation of organelle organization, neuron projection development	SMAD binding, protein serine/threonine kinase activity, transcription regulator activity, nucleic acid binding, ion binding
hsa-miR-655	MI0003677	14q32.31	14:101049550-101049646	B	hsa-miR-655-5p	MIMAT0026626	regulation of intracellular signal transduction	protein binding
					hsa-miR-655-3p	MIMAT0003331	positive regulation of translational initiation, positive regulation of protein autophosphorylation, platelet-derived growth factor receptor signaling pathway, smooth muscle cell differentiation, regulation of protein deacetylation, endocardial cushion development, artery morphogenesis	mRNA 3'-UTR binding, GTPase activator activity, DNA-binding transcription repressor activity, RNA polymerase II-specific, ubiquitin protein ligase activity, DNA-binding transcription activator activity, RNA polymerase II-specific, chromatin binding
hsa-miR-487a	MI0002471	14q32.31	14:101052446-101052525	B	hsa-miR-487a-5p	MIMAT0026559	neuromuscular junction development, hormone-mediated signaling pathway, cellular response to steroid hormone stimulus, transcription initiation from RNA polymerase II promoter, actin cytoskeleton organization, negative regulation of developmental process	RNA polymerase II transcription coactivator binding, nuclear receptor activity, enzyme binding
					hsa-miR-487a-3p	MIMAT0002178	regulation of cellular process	-
hsa-miR-382	MI0000790	14q32.31	14:101054306-101054381	B	hsa-miR-382-5p	MIMAT0000737	nervous system development	-
					hsa-miR-382-3p	MIMAT0022697	detection of chemical stimulus involved in sensory perception	binding
hsa-miR-134	MI0000474	14q32.31	14:101054687-101054759	B	hsa-miR-134-5p	MIMAT0000447	-	-
					hsa-miR-134-3p	MIMAT0026481	positive regulation of smoothened signaling pathway, blood vessel development, anatomical structure formation involved in morphogenesis, neurogenesis, cell development, regulation of cellular metabolic process, regulation of macromolecule metabolic process	protein binding
hsa-miR-668	MI0003761	14q32.31	14:101055258-101055323	B	hsa-miR-668-5p	MIMAT0026636	-	-
					hsa-miR-668-3p	MIMAT0003881	regulation of neuron projection development, regulation of anatomical structure morphogenesis, positive regulation of developmental process, regulation of cell differentiation, regulation of intracellular signal transduction, regulation of transcription by RNA polymerase II, cellular protein modification process	transferase activity, protein binding
hsa-miR-485	MI0002469	14q32.31	14:101055419-101055491	B	hsa-miR-485-5p	MIMAT0002175	regulation of small GTPase mediated signal transduction, anion transport, regulation of biological quality, negative regulation of cellular process, regulation of primary metabolic process	secondary active transmembrane transporter activity, protein binding
					hsa-miR-485-3p	MIMAT0002176	homophilic cell adhesion via plasma membrane adhesion molecules, cellular protein localization, negative regulation of nucleobase-containing compound metabolic process, nervous system development	ion binding, protein binding
hsa-miR-323b	MI0014206	14q32.31	14:101056219-101056300	B	hsa-miR-323b-5p	MIMAT0001630	dendritic spine morphogenesis,	frizzled binding, protein tyrosine kinase activity, sodium ion transmembrane transporter activity, voltage-gated ion channel activity, ubiquitin protein ligase binding, protein kinase binding, ATP binding
					hsa-miR-323b-3p	MIMAT0015050	-	-
hsa-miR-154	MI0000480	14q32.31	14:101059755-101059838	B	hsa-miR-154-5p	MIMAT0000452	regulation of intracellular protein transport, chromatin organization, cellular protein modification process, positive regulation of cellular process,	-
					hsa-miR-154-3p	MIMAT0000453	biological regulation	-
hsa-miR-496	MI0003136	14q32.31	14:101060628-101060649	B	hsa-miR-496	MIMAT0002818	-	metal ion binding
hsa-miR-377	MI0000785	14q32.31	14:101062050-101062118	B	hsa-miR-377-5p	MIMAT0004689	negative regulation of execution phase of apoptosis, regulation of signaling, regulation of cell communication	binding
					hsa-miR-377-3p	MIMAT0000730	regulation of microtubule motor activity, Wnt signaling pathway, calcium modulating pathway, positive regulation of blood vessel endothelial cell migration, histone modification, axonogenesis, positive regulation of cellular catabolic process, blood vessel morphogenesis, modulation of chemical synaptic transmission	Hsp90 protein binding, mRNA binding, phosphoric ester hydrolase activity, RNA polymerase II cis-regulatory region sequence-specific DNA binding, DNA-binding transcription factor activity, RNA polymerase II-specific, enzyme binding,

hsa-miR-541	MI0005539	14q32.31	14:101064495-101064578	B	hsa-miR-541-5p	MIMAT0004919	regulation of nucleobase-containing compound metabolic processregulation of macromolecule metabolic process, detection of chemical stimulus involved in sensory perception	RNA binding, metal ion binding, protein binding
					hsa-miR-541-3p	MIMAT0004920	SMAD protein signal transduction, cognition, regulation of secretion, positive regulation of transcription by RNA polymerase II, regulation of multicellular organismal process, system development, regulation of signal transduction, detection of chemical stimulus involved in sensory perception of smell	growth hormone receptor binding, enzyme binding
hsa-miR-409	MI0001735	14q32.31	14:101065300-101065378	B	hsa-miR-409-5p	MIMAT0001638	-	-
					hsa-miR-409-3p	MIMAT0001639	central nervous system neuron differentiation, forebrain development, vasculature development, negative regulation of transcription, DNA-templated, detection of chemical stimulus involved in sensory perception	protein binding
hsa-miR-412	MI0002464	14q32.31	14:101065447-101065537	B	hsa-miR-412-5p	MIMAT0026557	-	-
					hsa-miR-412-3p	MIMAT0002170	positive regulation of cellular component organization, positive regulation of cellular metabolic process	RNA polymerase II cis-regulatory region sequence-specific DNA binding, DNA-binding transcription factor activity, RNA polymerase II-specific
hsa-miR-369	MI0000777	14q32.31	14:101065598-101065667	B	hsa-miR-369-5p	MIMAT0001621	-	-
					hsa-miR-369-3p	MIMAT0000721	dentate gyrus development, sequestering of extracellular ligand from receptor, positive regulation of translational initiation, neuroblast proliferation, forebrain neuron development, ventricular septum morphogenesis, hippo signaling, positive regulation of BMP signaling pathway, cyclic nucleotide metabolic process, cyclic-nucleotide-mediated signaling, ureteric bud morphogenesis	DNA-binding transcription activator activity, RNA polymerase II-specific, DNA-binding transcription factor binding, protein domain specific binding, RNA polymerase II cis-regulatory region sequence-specific DNA binding, zinc ion binding
hsa-miR-410	MI0002465	14q32.31	14:101065912-101065991	B	hsa-miR-410-5p	MIMAT0026558	peptidyl-amino acid modification	-
					hsa-miR-410-3p	MIMAT0002171	negative regulation of SMAD protein complex assembly, positive regulation of deacetylase activity, positive regulation of cardiac muscle cell proliferation, regulation of vasculogenesis, cell proliferation in forebrain, mRNA destabilization, canonical Wnt signaling pathway, response to fluid shear stress, regulation of cAMP-mediated signaling	SMAD binding, PDZ domain binding, mRNA binding, DNA-binding transcription activator activity, RNA polymerase II-specific, chromatin binding, transcription factor binding, transferase activity
hsa-miR-656	MI0003678	14q32.31	14:101066724-101066801	B	hsa-miR-656-5p	MIMAT0026627	regulation of postsynapse organization, peripheral nervous system development, response to acid chemical, positive regulation of neuron projection development, central nervous system neuron differentiation, regulation of endothelial cell migration, developmental growth, regulation of epithelial cell proliferation, axon guidance	guanyl-nucleotide exchange factor activity, DNA-binding transcription activator activity, RNA polymerase II-specific, RNA polymerase II cis-regulatory region sequence-specific DNA binding, enzyme binding, metal ion binding
					hsa-miR-656-3p	MIMAT0003332	-	protein binding
